# In-vitro accuracy of the virtual patient model with maxillomandibular relationship at centric occlusion using 3D-printed customized transfer key

**DOI:** 10.1038/s41405-025-00303-1

**Published:** 2025-01-31

**Authors:** Anh Ho-Quynh Nguyen, Nam Cong-Nhat Huynh, Oanh Ngoc-Hoang Nguyen, Nhat Dinh-Minh Nguyen, Hai Hoang Phan, Jong-Eun Kim, Gan Jin, Khanh Hung Nguyen, Hung Trong Hoang

**Affiliations:** 1 Faculty of Dentistry, University of Medicine and Pharmacy at Ho Chi Minh City, Ho Chi Minh City, Vietnam; 2 Department of Prosthodontics, Yonsei University College of Dentistry, Seoul, Korea

**Keywords:** Oral anatomy, Occlusion, Prosthetic dentistry

## Abstract

**Objective:**

This study aimed to create a 3D-printed customized transfer key and evaluate the accuracy of the virtual patient model with maxillomandibular relationship at centric occlusion using the transfer key.

**Methods:**

A 3D-printed transfer key was designed, combining facial and intraoral (IOS) scans. The design included components that recorded the 3D upper and lower arch at centric occlusion. The virtual patient model image was generated in-vitro using a phantom head with soft tissue simulation. Accuracy was assessed by superimposing the 3D scans with reference CBCT images and analyzing trueness and precision using root mean square (RMS) deviations.

**Results:**

The transfer key included an intra-oral part that acts as an anterior deprogrammer to record the relationship of two dental arches at centric occlusion (CO) and an extra-oral with a rotatable cross-shaped design with two arms for locating the facial midline and the two pupils connecting line. Superimposition demonstrated high trueness (RMS: 0.51 mm for the arch regions, 0.69 mm for the whole head region, 0.85 mm in the face region) and precision (RMS: 0.41 mm for the arch regions, 0.52 mm for the entire head, 0.63 mm in the face region) significantly (*p* < 0.05). Minimal deviations were observed in critical areas, including the tooth and lip position, indicating that the virtual patient model was closely aligned with the CBCT reference. The dental arches achieved the highest accuracy, while slight deviations were noted in the facial regions.

**Conclusions:**

The 3D-printed customized transfer key effectively enhanced the virtual patient model’s accuracy, surpassing traditional trueness and precision methods. This novel approach offers a streamlined, patient-friendly solution for digital dental workflows.

## Introduction

Comprehensive dental treatment necessitates careful consideration and advanced planning, particularly concerning occlusal design, before initiating any procedures. The goal in such cases is to restore functional and biological efficiency, ensuring that the teeth, periodontal structures, masticatory muscles, and temporomandibular joint (TMJ) mechanisms all work in synchronized harmony. As such, a thorough evaluation of the relationship between the dental arches and other components of the masticatory system is critical for successful outcomes, from diagnosis through to the completion of treatment. In many cases, especially complex and comprehensive cases, we do not have enough time to work and check the information in the dental chair. Hence, we expect to create a set of data that most closely simulates the patient’s actual condition, including teeth and dental arches about bones and faces. Two critical references in this simulation include the 3D position of the upper dental arch in the skull and the maxillomandibular relationship [1, 2]. Traditionally, we perform this by taking impressions, pouring plaster casts, and mounting casts with a facebow and articulator. Accurately mounting the mandibular arch (lower jaw) on an articulator is essential for diagnosing and planning treatments. This process involves determining whether to position the mandible seats in maximal intercuspal position (MIP) or centric occlusion (CO). In such cases, according to The Glossary of Prosthodontic Terms (v.10th, 2023) centric relation (CR) is a maxillomandibular relationship in which the condyles articulate in the anterior-superior position against the posterior slopes of the articular eminences while CO is the teeth position when the TMJ is at centric relation (CR), wherein the condyles articulate against the posterior slopes of the articular eminences in an anterior-superior posture. The mandible can only move fully rotating, providing a repeatable, therapeutically valuable reference position [3]. Although ideally, MIP should coincide with CO, in most cases, there is a discrepancy between CO và MIP. Selecting between MIP and CO for mandibular mounting should be tailored to the patient’s specific clinical situation. MIP is generally appropriate for individuals with stable occlusion and no temporomandibular joint (TMJ) disorders. Conversely, in cases involving occlusal discrepancies or TMJ issues, utilizing CO can offer a more precise representation of the jaw relationship, thereby enhancing treatment planning [4].

On the other hand, maxillary mounting is a critical step, as its accuracy directly influences the success of subsequent stages. This step establishes the upper occlusal plane about the hinge axis. However, even using a facebow does not provide complete facial information, making it difficult to achieve a comprehensive plan in terms of both aesthetics and function. In contemporary dentistry, noticing and focusing on facial-driven dental treatment is crucial. Therefore, simulating the occlusion in a 3D relationship with joint and facial landmarks is vital [5].

Nowadays, with the continuous development of digital dentistry, creating a virtual patient model that visually simulates the face and occlusion has become a reality. The digitization of dental arches, as well as the digitization of faces, have gradually become popular. In the digital workflow, virtual mandible mounting, which simulates the maxillomandibular relationship, can be easily obtained with an intra-oral scanner (IOS) [6, 7]. However, digitized images of the face and dental arches can be combined in a simple, convenient way for doctors and patients. A high-accuracy virtual patient model is still a challenge [8, 9]. Although recent studies have demonstrated the potential of using a virtual facebow and facial scan files to create a virtual model, the accuracy of 3D maxillary mounting remains a limitation that should be improved. Previous studies have also indicated some suggestions for designing the transfer device to reduce data deviation and enhance patient comfort [10]. However, a lack of studies combine exactly the facial data and dental arch at CO when the TMJ is in CR. Here, we aimed to develop a simple customized transfer key that facilitated upper dental arch virtual mounting using a face scanner while recording the 3D position of the lower arch at CO with an intraoral scanner. We then evaluated the virtual patient model’s accuracy (trueness and precision) with a maxillomandibular relationship at CO in-vitro.

## Materials and methods

### Study design

**Fig. 1:**
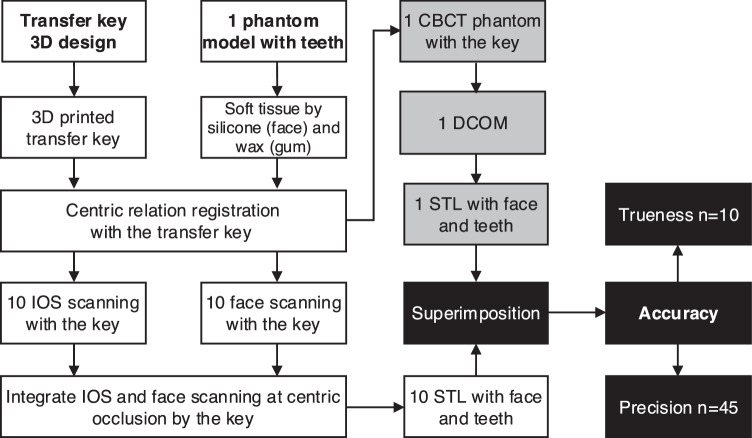
Study design. The transfer key was designed and 3D fabricated followed by phantom head preparation with soft tissue simulation. CBCT, face scan data and IOS scan data were obtained, and superimposition was performed to evaluate accuracy.

In the in-vitro study, the subject was a phantom skull with soft tissue and full dental arches. A 3D-printed customized transfer key was designed and developed. The key was used for the integration of facial scan data and intra-oral scan data, facilitating the creation of a comprehensive virtual phantom patient model. The accuracy of the virtual model using the superimposition method (Fig. 1), according to ISO 5725-1:2023 (Accuracy - trueness and precision - of measurement methods and results), including the trueness with one reference image compared to 10 experimental images (*n* = 10 pairs) and the precision with each pair in 10 experimental images (*n* = C_10_^2^ = 45 pairs) [11, 12]. Our previous studies applied this sample size standard for the investigation of accuracy [6, 7, 12, 13].

### Transfer key design and 3D printed manufacture

The transfer key was designed in Autodesk Fusion 360 (Education version, Autodesk, USA). After optimizing the design, the final version of the transfer key was then printed by Formlab Form 3B 3D printer (Formlab, USA) with Laser-Powered Stereolithography (SLA) technology Low Force Stereolithography (LFS) printer engine using WhiteResin resin (FLGPWH04) composing urethane dimethacrylate and methacrylate monomers. No biological exposure limit was noted for the ingredients regarding the biological limit values. The layer thickness was set to 0.1 mm, and the product was processed according to the manufacturer’s instructions [13].

### Phantom head prepare

**Fig. 2:**
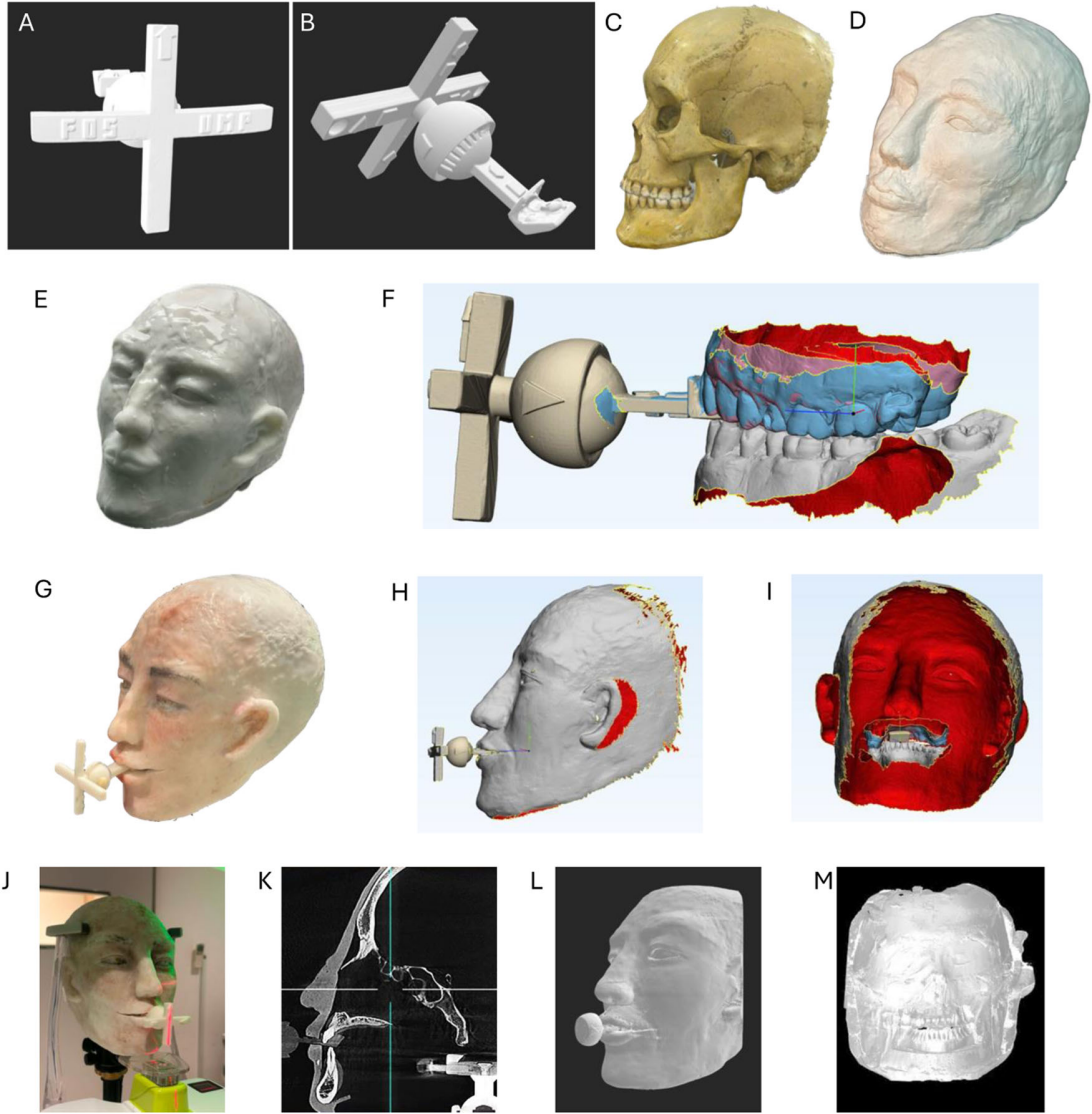
Transfer key, Phantom head, and 3D image preparation. **A** 3D image of the scanned transfer key from the front side. **B** 3D image of the scanned transfer key from the lateral side. **C** Adult skull model. **D** Skull model covered by clay. **E** Skull model covered silicone. **F** Teeth and bite scans at centric occlusion using transfer key. **G** The finishing Phantom head with colored silicone cover and transfer key. **H** 3D image of the scanned phantom head with transfer key. **I** 3D image of the scanned phantom head from behind with the full arches at centric occlusion. **J** Phantom head during CBCT capture. **K** CBCT image of the phantom head. **L** 3D image of converted CBCT image of the phantom head from outside. **M** 3D image of converted CBCT image of the phantom head from behind with the full arches at centric occlusion.

This study used a human adult skull from the Department of Oral Radiology (University of Medicine and Pharmacy at Ho Chi Minh City) with full arches at Angle I occlusion. To design the skin model, we used modeling clay for sculpture to create the face shape with general anatomical landmarks such as ear, eyes, nose, and lips. Then, the silicone mold was cast from the sculpted skin model. The liquid silicone and catalyst (Elite Double 22 Normal, Zhermack, Italy) were mixed with an earthy orange color for skin tone according to the manufacturer’s instructions for preparing the silicone mixture. The silicone mixture was poured into the mold, and the air bubbles were removed after complete polymerization for 24 h at room temperature. The silicone layer was gently removed from the mold, trimming the details on the silicone skin, including eye, nose, and mouth cavities, which were adjusted to ensure a good fit with the skull. Finally, silicone color was used to create details for the skin layer, simulating natural facial skin and covering the back surface with medical glue. The completed silicone skin layer was installed on the skull, and fit testing was done in a static state or when the lower jaw was moving (Fig. 2C–E). The artificial gingiva was created using dental wax and sculpted to mimic the anatomical structure of the gums.

### Centric relation establishment and key setting up

The simulated CR of the phantom head was established, and the CO was recorded using the insert part, which acted similarly to a Lucia jig. The disc was unnoticed in this in-vitro design. We manually guided the mandible to the position where the left and right condyles of the skull sit in the center of the fossa to simulate the centric relation position. At that time, only the lower teeth were in contact with the surface of the bite plan of the key; the posterior teeth were completely open [2]. The contact point of the lower incisors on the insert’s bite plan was marked with articulator paper. After guiding the lower jaw to the correct contact position on the bite plan, the self-cure resin was used to create two palatal occlusal locks on the first premolars and molars on both the left and right sides. The receptacle part was rotated until one arm aligned with the facial midline while the other was parallel to the line connecting the two pupils (Fig. 2G, H). The spherical swivel joint connecting the insert part and the receptacle part of the transfer key was then secured. An experienced occlusion instructor conducted this entire procedure. The fixed key was then scanned with an in-lab scanner (3Shape, Denmark) to get the STL file of the key.

### Scanning data acquisition

The phantom head was mounted on the dental simulation unit, and intra-oral data were obtained by the IOS TriOS4 scanner (wireless version, 3Shape, Denmark) using a standard scan strategy recommended by 3Shape. The upper was scanned twice, one with and one without the insert part of the key. Bite scan data was recorded using the transfer key with the support of two palatal occlusal locks at CO. The obtained scanning files were all stored in STL format. IOS-STL files were exported for integration (Fig. 2F). Intra-oral and face scanning following data merging was repeated 10 times.

For face scanning, the phantom head was kept in a natural position with eyes facing forward, the lines connecting the two pupils, and the lines connecting the corner of the eyes to the top of the ears parallel to the floor. The bite was placed at centric occlusion using the transfer key. MetiSmile face scanner (Shining3D, China) with resolution: data acquisition camera 1.3 Mega Pixel, HD texture camera 5.0 Mega Pixel, published accuracy 50μm, optical field: 500 mm, FOV 210*270 mm, was set to handheld mode according to the manufacturer’s instructions. Computer systems and software were included with the scanner. In brief, the scanner was held at a distance of about 50 cm from the face surface with optimal brightness adjustment, scanning from left to right, going around the entire face, then scanning from the chin to the nose over the head, from under the left ear to the head over the right ear. The transfer key was also recorded during the scanning process. The scanning process was monitored on the computer output image, ensuring that the entire face, ears, chin, forehead, and transfer key were scanned. STL files were then exported for integration (Fig. 2H).

### IOS and face scan integration to create intergrated virtual patient models

**Fig. 3:**
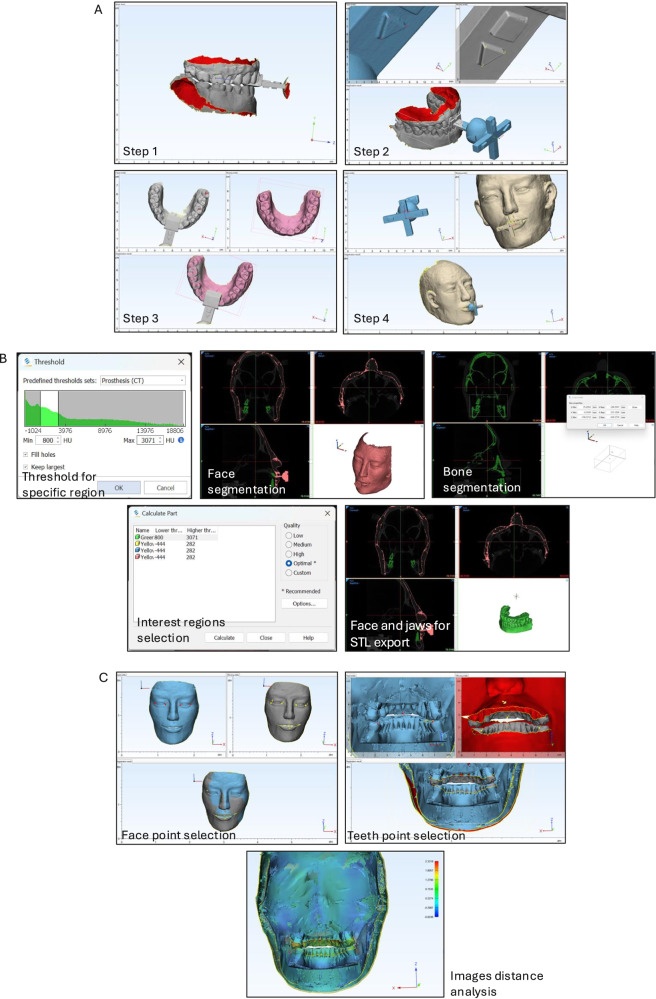
The integration, STL conversion, and superimposition procedure. **A** The integration of face and dental arches scans at centric occlusion. The step 1–4 were described in Materials and Methods. **B** The conversion from DCOM to STL procedure. After the segmentation of specific regions by threshold selection, face, and jaws were retained for STL export. **C** The superimposition procedure of the virtual patient model data. Two images were superimposed by point selecting and then auto-global registration. Then the distance between two surfaces after convolution was analyzed and calculated.

All the STL files were then processed in 3-Matic Research software (v.13, Materialize NV, Belgium). Then, the IOS files of both jaws were imported (step 1), and the upper jaw’s IOS was aligned with the in-lab-scanned data of the key using overlapping reference points on the extent horizontal bar of the intra-oral part of the key (step 2). The upper jaw’s IOS without the insert was then aligned using the four sharpest cusp reference points at teeth 14, 24, 18, and 28 (step 3). The face scan data was also imported and aligned with in-lab-scanned data of the key using its extra-oral part. Once the alignment was completed, in-lab-scanned key and upper jaw data with key data were removed. The transfer key in the face scan data was also trimmed (step 4). Finally, the face scan and two dental arches were merged into a single STL file to create a integrated virtual patient model. This detailed protocol ensured precise 3D modeling of the craniofacial structure for virtual applications (Figs. 2F–I and 3A).

### CBCT image obtaining and STL conversion for reference virtual patient model

The phantom head was captured by CBCT Rainbow (Dentium, Korea) with CMOS sensor, 0.5 mm focal point, 100–300 μm voxel size, and FOV up to 16 × 18 cm that can capture the full-face area. The imaging process was performed according to the manufacturer’s instructions, using the FOV of the model’s entire face, teeth, and transfer key (Fig. 2J). In the CBCT image, we could notice the layers of teeth, bone, silicon for skin, and part of the transfer key. The gum recreated by dental wax was not captured (Fig. 2K).

One output file was stored in DCOM format and converted to STL format using Mimics Research software (v.21, Materialize NV, Belgium) according to the previous studies [14] (Fig. 3B). The generation of an STL file from DICOM data commenced with importing the DICOM dataset, consisting of multiple image slices. All layers were subsequently merged to create a cohesive dataset for processing. A mask was then generated, with threshold values applied to segment the specific region of interest, encompassing the facial and jaw structures while excluding the osseous areas. Tools such as split masks, edit masks, and crop masks were employed to refine the selection, enabling zoomed and precise isolation of the targeted soft tissues while disregarding non-essential areas. The Mask 3D Preview mode facilitated a simulated visualization of the 3D reconstruction for validation purposes. Following successful segmentation, the mask was converted to a 3D mesh by calculating parts into discrete objects. Extraneous small shells were filtered out to enhance model fidelity, and the final STL file was exported. Finally, an STL file that contained soft tissue and two arches was generated from the DCOM file. This STL was then used as a reference for the superimposition stage (Fig. 2L, M). This workflow yielded an accurate and focused 3D representation of the specified anatomical region, suitable for further analysis and application.

### Superimposition process

The method of superimposing 3-D images from STL files was used using 3-Matic Research software as in our previous study [12]. The gum of experimental images was clear and consistent with the CBCT image. The files were also trimmed, and artifacts were removed for the final analysis. In the first step, 7 points on the reference image (1 reconstructed STL file from CBCT) and the corresponding points in the survey images (1 in 10 STL files of experimental images) were selected, including 5 face points: Ex (Exocanthion left and right), En (Endocanthion, left and right); 3 teeth points: middle of the incisal edge of tooth 11, distolingual cusp apex of teeth 38, 48 for the whole face and teeth superimposition. The software then analyzed and superimposed two images to make the distance between these pairs of points minimal. After convolution by point, full-surface convolution was performed automatically to ensure the minimum distance between two surfaces. In the second step, the distance between two surfaces after convolution was analyzed and calculated, showing a color scale in which the blue area: the experimental image > reference image; green area: no difference between the two images; and red area: experimental image < reference image. We calculated the minimum, maximum, and mean deviations and the root mean square (RMS) for each pair of images. RMS value demonstrated the data-point clouds of surfaces and showed how two images deviated from zero as low RMS indicated the good agreement of two 3D images (Fig. 3C). We compared both the face and teeth scans of the integrated virtual patient models to the CBCT reference virtual patient model of the exact position of the upper jaw and lower jaw at CR.

### Statistical analysis

GraphPad Prism (v.10, GraphPad Software, USA) was used for the statistical analysis and plots. The data were shown as median and interquartile or mean ± standard deviation (SD). The normality was verified by the Shapiro–Wilk test. One-way ANOVA with Tukey post hoc test was applied for multiple comparisons and correction. *P* values < 0.05 were regarded as statistical significance. Achieved power ≥80% was calculated by G*Power (v3.1) [15] (Supplementary Table S1).

## Results

### Simple 3D-printed customized transfer key

We successfully designed a simple and affordable transfer key, including two components: an intra-oral part (insert part) that acts as an anterior deprogrammer to record the relationship of two dental arches at CO and an extra-oral (receptacle part) with a rotatable cross-shaped design with two arms for locating the facial midline and the two pupils connecting line. The rotating part and the bite plan were separated and connected via a spherical swivel joint with a pin and slot. Embossed shapes were strategically added in certain areas to serve as anchors (Fig. 2A, B).

### The trueness of the integrated virtual patient model

**Fig. 4:**
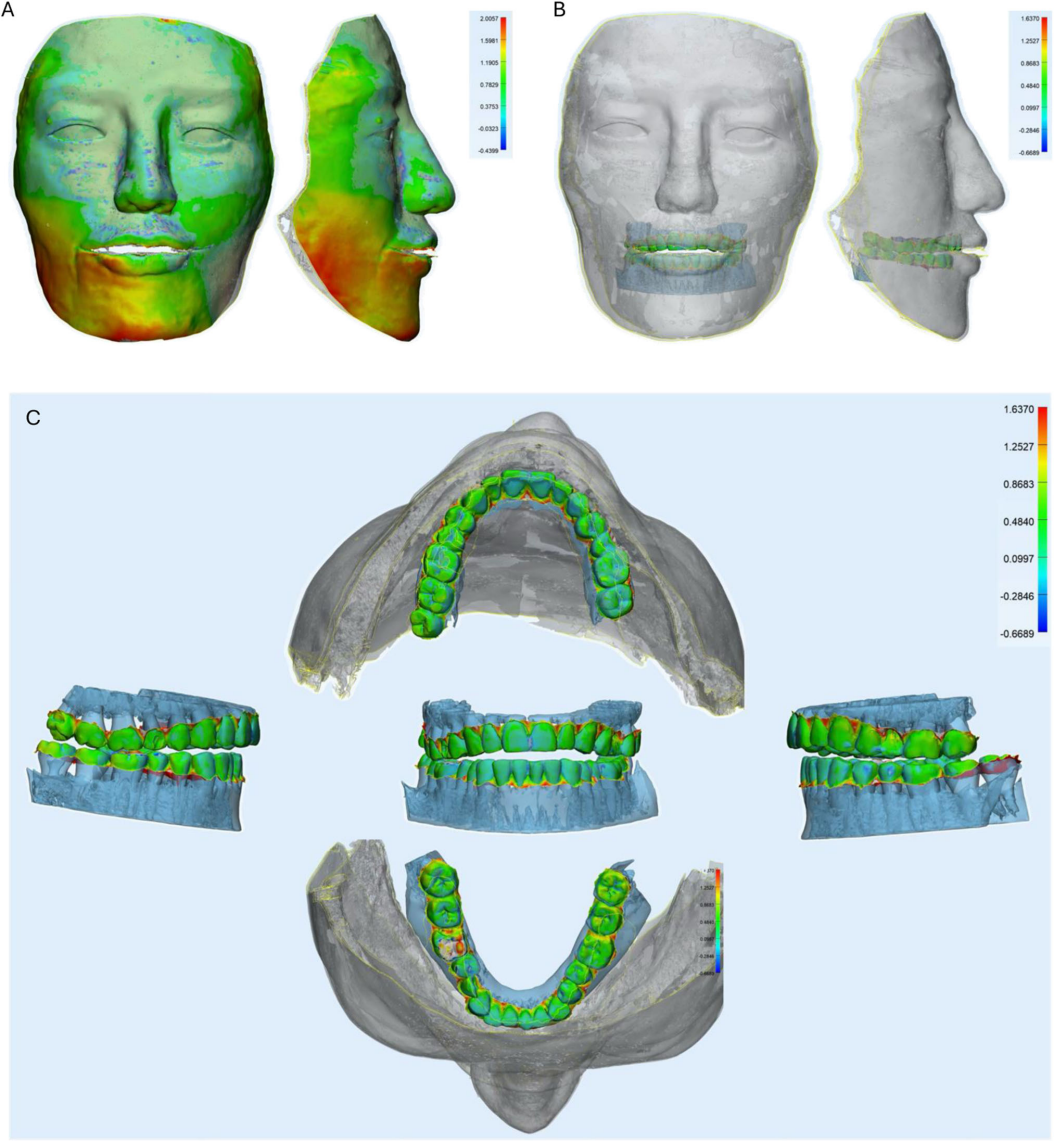
The superimposition of the trueness. **A** Face analysis of superimposition from the front and lateral side. **B** Arches analysis of superimposition from the front and side of the face. **C** Arches analysis of superimposition at 5 standard positions (front, left/right lateral sides, upper and lower arches). The color scale was in mm.

After successfully constructing a virtual phantom head with the face and dental arches at CO, we investigated the accuracy of this model by using the 3D version from the CBCT image as the reference. After superimposing the total virtual phantom head, we analyzed the whole area, the face, and the teeth deviation for clear demonstration. The trueness showed a high agreement in CBCT 3D reference and experimental images in general. The most different areas were the lateral cheek and the chin, with a disparity of around 2 mm, where there was no difference in the upper, middle face, and lip areas (Fig. 4A).

For teeth superimposition, high identity was observed in both the upper and lower arches, showing in front, lateral, and occlusion views (Fig. 4B, C). One artifact area during the 3D reconstruction of CBCT on tooth 16 was clean (gray color). Some extending areas (red color) were located in the gum papilla due to the trimming process (Fig. 4C).

Min values of the deviations were from −0.87 to −0.48 mm, while the max values were from 1.85 to 2.66 mm, and the mean deviations were 0.18–0.54 mm. RMS value indicated the agreement of images expressed 0.51 mm in the arche region, 0.85 mm in the face region, and 0.69 mm in the whole head region (Fig. 6A). There were significant differences in dental arches to the entire head and the face in which the arches the highest trueness and the face was lowest one.

### The precision of the integrated virtual patient model

**Fig. 5:**
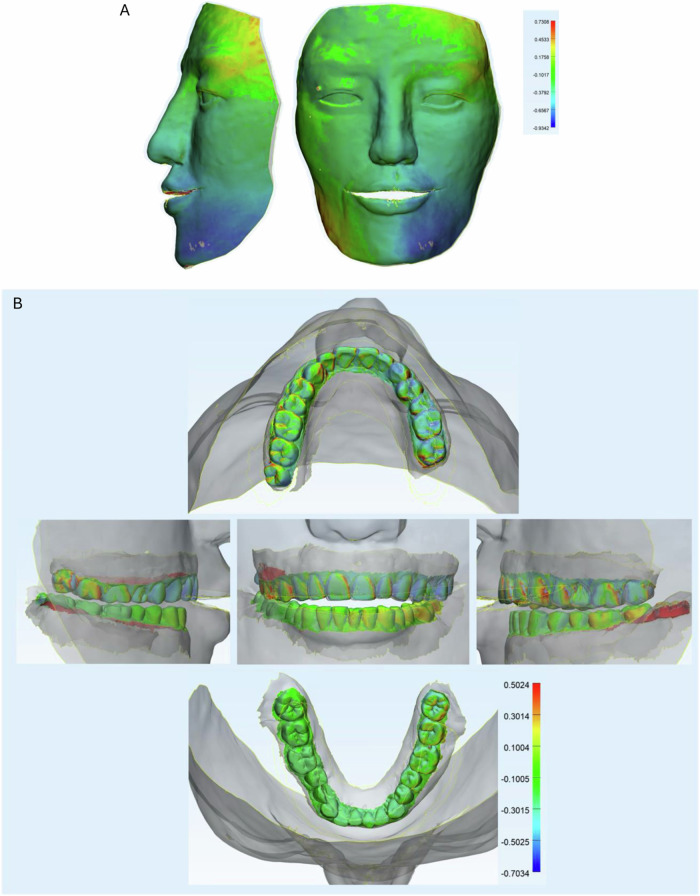
The superimposition of the precision. **A** Face analysis of superimposition from the front and lateral side. **B** Arches analysis of superimposition at 5 standard positions (front, left/right lateral sides, upper and lower arches). The color scale was in mm.

The superimposition of precision showed a high agreement between images. However, the lower cheek and mandible angles expressed a higher difference, similar to the trueness. No change in the upper and middle faces and lip areas (Fig. 5A). Arches superimposition of the precision demonstrated the high shift in the interproximal and distal areas, especially in the upper arches (Fig. 5B).

**Fig. 6:**
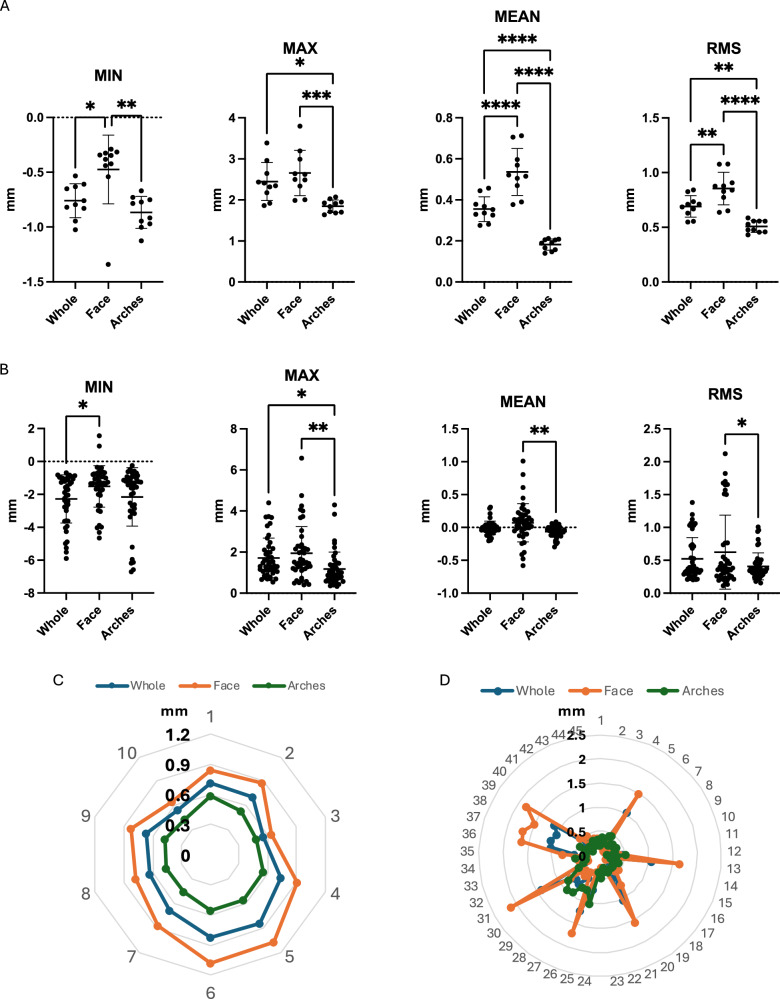
Comparison of trueness and precision values of superimposition in the whole, face, and arches images. **A** The trueness’s min, max, mean, and RMS (root mean square) deviation values, *n* = 10. **B** The precision’s min, max, mean, and RMS (root mean square) deviation values, *n* = 45. **C** Radar plot of RMS value of the trueness in 3 groups. **D** Radar plot of RMS value of the precision in 3 groups (*p < 0.05, **p < 0.01, ***p < 0.001, ****p < 0.0001).

In the precision, there was more deviation in which min values were from −2.23 to −1.52 mm, while the max values were from 1.18 to 1.94 mm, and the mean deviations were from −0.06 to 0.07 mm. However, high agreement was expressed in RMS as 0.41 mm in the arch regions, 0.63 mm in the face region, and 0.52 mm in the whole head region (Fig. 6B). There were significant differences in arches to the entire head and the face in which the dental arches got the highest precision and the face was lowest one.

In summary, the trueness and precision of arches were in the highest agreement (0.41–0.51 mm). Face got an accuracy of 0.63–0.85 mm with the highest deviation, and the whole virtual phantom head expressed 0.52–0.69 mm of accuracy (Fig. 6C, D).

## Discussion

Our study developed a customized transfer key and evaluated its trueness and precision in the virtual patient model. More than the correct three-dimensional alignment of the upper and lower jaw relationships is required to achieve optimal treatment outcomes, particularly in comprehensive functional and aesthetic dental restoration. Accurately replicating the three-dimensional position of the maxilla relative to the cranial base, particularly concerning the temporomandibular joint socket, is also essential. This involves creating a virtual patient model that closely approximates the anatomy in digital dentistry. Our findings indicate that developing a digital virtual head model with relatively high accuracy is promising, as demonstrated by the precision and trueness of 0.69 mm. Additionally, the 3D position of dental arches in centric relation can be reproducibly recorded with an average trueness of 0.51 mm and precision of 0.41 mm. In a comparable study, Li et al. reported the reproducibility of maxillary 3D position with an average trueness of 1.14 mm and precision of 1.09 mm [16]. However, their study collected facial scan data using smartphone scanners, which may affect research outcomes and introduce more significant inaccuracies. Based on our findings and previously reported data [17, 18], we recommend using industrial-grade 3D scanners to acquire more precise facial data. In the digital workflow, 3D facial scanners performed comparably to traditional facebow records for virtual dental cast mounting [19]. For the highest accuracy in cases requiring precise alignment of the jaw relative to the cranial base, combining a transfer device with a facial scanner is likely to yield optimal results [1, 20, 21]. The devices play a crucial role in the integration of facial data and IOS data.

The primary advantage of the transfer key developed in our study is its ability to simultaneously record two critical references for comprehensive occlusal simulation: (1) the relationship between maxillary and mandibular dental arches and (2) the three-dimensional relationship between occlusion and the cranial base. Our concept employs an anterior deprogrammer with an extra-oral extension that enables simultaneous capture of the maxillary–mandibular relationship at the centric occlusion and the integration of both facial and intraoral scan data. The transfer key provides adjustments for the facial midline and interpupillary line using the extraoral part, which was shown on the facial scan data. The intra-oral part of the key was scanned during IOS scanning, which also contained the upper dental arch. Therefore, the occlusal plane information was also collected and transferred to the virtual phantom model by integration of scanned data.

The intermaxillary relationship in centric relation is traditionally recorded in the analog workflow using methods such as cast articulation, maximum intercuspation (MIP), or Lucia jig combined with a bite index for CO-CR recording. Additionally, a facebow can be used to localize the maxilla’s 3D position relative to the reference planes and terminal hinge axis movement [2]. Several studies emphasize the importance of facebows in dental treatment planning [18, 22]. In a digital workflow, the first reference can be recorded efficiently using an intraoral scanner with or without an anterior deprogrammer, depending on the case requirements. The second reference is more complex, and current methods involve superimposing IOS data with two-dimensional data, such as facial photographs or cephalometric X-rays, or three-dimensional data, such as CBCT scans, or by combining a virtual facebow and facial scan data [1]. The hinge axis and third point of reference are important in the traditional facebow transfer data to simulate the patient’s real condition. In digital workflow, by integrating the facial 3D scan data and intra-oral scan data, we could also create a virtual model that simulates the real patient. The mounting procedure may be slightly different between analog and digital workflow, although the final objects all simulate the closest patient’s real condition. The intra-oral part of the key was scanned during IOS scanning, which also contained the upper dental arch. Therefore, the occlusal reference plane information was also collected and transferred to the virtual model by integration of scanned data. In this study, we compared the difference between CBCT data and a combination of full-facial data and IOS data. Our study applied the segmentation strategy as the previous study with evidenced accuracy [14, 23]. Moreover, DCOM data in this study was the available reference that involved both face and jaw information at centric occlusion. In fact, a study also used CBCT data as a reference when investigating the accuracy of virtual facebow records [16].

Our transfer key is designed to capture these two essential data points accurately. The intraoral insert component of our transfer key is inspired by the Lucia jig, making it easy to use, operate, and familiar to many practitioners. This component acts as an anterior deprogrammer, facilitating simple and effective recording of the centric relation (CR) position, which is critical in dental practice, especially in complex cases such as full-mouth rehabilitation and orthodontics. By mounting on the articulator in centric relation, centric occlusion can be assessed or adjusted as necessary. With its adjustable arm system for midline and pupil alignment, the extraoral receptacle component offers adaptability across diverse facial anatomies, further enhancing its utility in clinical applications. Incorporating 3D printing technology to fabricate customized transfer keys represents a significant advancement in the digital workflow for occlusal analysis on the virtual patient model. By combining this transfer key with IOS and facial scans, we achieved precision and trueness comparable to, and potentially exceeding, that of traditional facebow techniques, as supported by prior studies on the kinematic facebow and virtual facebow [24, 25]. The accuracy of the virtual patient model was validated by superimposing 3D images generated from IOS and facial scans onto CBCT-derived STL files. This analysis confirmed high levels of trueness and precision, particularly in the facial and dental regions critical for occlusal adjustments and restorations. Given the reliance on accurate jaw positioning in treatments such as orthodontics, full-mouth reconstructions, and TMJ-related therapies, the improved fidelity of virtual mounting with transfer key holds substantial clinical relevance. Additionally, the large field-of-view CBCT in the present study was used as the reference to evaluate the trueness of 3D images generated from the combination of face and teeth scans via transfer key at centric occlusion. In clinical situations, we can use only a face scanner and IOS with a transfer key to obtain the virtual patient model without CBCT. It reduces the usage of CBCT in unnecessary cases.

In-vitro study design is the standard method to investigate the accuracy of optical devices and 3D images to eliminate any potential bias in clinical situations. Many previous studies investigated the virtual facebow technique’s accuracy and used phantom models [1, 8, 20, 21]. However, this design, while controlled, may not fully replicate clinical complexities such as patient movement, CR position, and variations in soft tissue resilience. Additionally, exploring further refinements in the transfer key’s materials or design could enhance its durability and adaptability. In our study, limitations arose due to the partial fixation of the mobile edge of the silicone coating in the mandibular angles of the skull model. Although partially fixed, this edge could still move to some extent, contributing to the observed deviations in the face scan data. Furthermore, the alveolar bone and interproximal contact areas were covered with dental wax to simulate gingiva for improved IOS scanning. However, the wax was not represented on the CBCT and 3D reference images and was trimmed during superimposition. Inaccurate trimming in some areas of the gingival papilla was noted, as highlighted in red. Our present study did not integrate the dynamic data, but only static data. These issues can be addressed in subsequent human studies to assess the transfer key’s clinical effectiveness. In the next studies, we continue to confirm our method’s accuracy in clinical and motion-tracking situations.

## Conclusion

Our study presents a novel, customized transfer key that enhances the accuracy and efficiency of virtual patient modeling with maxillomandibular relationship at centric occlusion. By integrating facial and intraoral scans with a 3D-printed transfer key, this approach provides a promising alternative to traditional methods, paving the way for more streamlined and patient-friendly digital dental workflows. Future studies should include in-vivo trials to assess the tool’s robustness and accuracy in dynamic clinical settings.

## Supplementary information

Supplementary table S1

## Data Availability

The datasets used and/or analyzed during the current study are available from the corresponding author upon reasonable request.
