# Supplementary material for: In-vitro accuracy of the virtual patient model with maxillomandibular relationship at centric occlusion using 3D-printed customized transfer key
Source: BDJ Open. 2025 Jan 31;11:8. doi: 10.1038/s41405-025-00303-1 (PMC11785937; doi:10.1038/s41405-025-00303-1)
Supplement: Supplementary file 1 — Supplementary table S1 [file 41405_2025_303_MOESM1_ESM.docx]

| **Trueness (n=10)** | **Comparison** | **p-value (power)** |
| --- | --- | --- |
| MIN | Whole vs. Face | 0.0187 (80%) |
|  | Face vs. Arches | 0.0012 (96%) |
| MAX | Whole vs. Arches | 0.0104 (98%) |
|  | Face vs. Arches | 0.0006 (100%) |
| MEAN | Whole vs. Face | <0.0001 (100%) |
|  | Whole vs. Arches | <0.0001 (100%) |
|  | Face vs. Arches | <0.0001 (100%) |
| RMS | Whole vs. Face | 0.0059 (87%) |
|  | Whole vs. Arches | 0.0021 (100%) |
|  | Face vs. Arches | <0.0001 (100%) |

**Supplementary table S1:** *P*-values of significant trueness comparison (p<0.05) and achieved power.
